# Filtered dataset of Irish energy performance certificates: A data-driven approach for enhanced building stock modelling

**DOI:** 10.1016/j.dib.2025.111281

**Published:** 2025-01-08

**Authors:** Kumar Raushan, Tomás Mac Uidhir, Marisa Llorens Salvador, Brian Norton, Ciara Ahern

**Affiliations:** a Dublin Energy Lab and Built Environment Research & Innovation Centre, Technological University Dublin, Ireland; b MaREI, the SFI Centre for Energy Climate and Marine, Ireland; c Energy Policy and Modelling Group, MaREI Centre, Environmental Research Institute, University College Cork, Lee Road, Cork, Ireland; d International Energy Research Centre, Tyndall National Institute, University College Cork, Cork, Ireland; e Irish Building Stock Observatory, Ireland

**Keywords:** Energy performance certificates, Data validation, Data-driven statistical methods, Dwelling energy assessment procedure, EPC database, Building energy rating, Irish housing stock

## Abstract

The data presented in this article supports the research publication “A data-driven standardised generalisable methodology to validate a large energy performance Certification dataset: A case of the application in Ireland” by Raushan et al. [1]. It provides the filtered Energy Performance Certificate (EPC) database for residential buildings in Ireland after applying rigorous data validation methods to remove erroneous entries, and outliers. EPCs contain valuable information about building energy efficiency and characteristics. The raw EPC database for Ireland is publicly accessible but contains over 1 million unfiltered entries with inconsistent and erroneous values that can skew analysis. This processed dataset enhances the quality and robustness of the EPC data for use in building stock modelling and research. The data is openly available in .CSV format along with the methodology used for processing the raw database, published in full Python scripts. Supporting notes and metadata explain the filtering process, experimental design, and content of 211 variables across four categories: Informational, form, envelope, and system. By publishing this standardised data-driven filtered EPC dataset, this research enables stakeholders, non-expert and expert alike, to leverage this higher quality input for characterising the Irish housing stock.

Specifications Table

**Table:** 

Subject	Engineering (General), Energy Performance Certification*.*
Specific subject area	Geometric, thermophysical, and system efficiency specification of Ireland's housing stock*.*
Type of data	Raw, Filtered, Processed
Data collection	An unfiltered (raw) EPC/BER database was acquired from the Sustainable Energy Authority of Ireland (SEAI). Python (supported version 3.8.8 is used to process this database and generate new filters. EPC data is available in public domain, online hosted by Sustainable Energy Authority of Ireland. Primary Data is collected manually by assessors using DEAP methodology and associated software [1].
Data source location	*Data is hosted online and available for download in raw, unfiltered .txt format from the SEAI* [2], https://ndber.seai.ie/BERResearchTool/ber/search.aspx
Data accessibility	Repository name: A generalisable data-driven filtering methodology for Energy Performance Certification databases. [3] Data identification number: 10.17632/hxpmt994js.1 Direct URL to data: https://data.mendeley.com/datasets/hxpmt994js/1
Related research article	K. Raushan, T. Mac Uidhir, M. Llorens Salvador, B. Norton, C. Ahern, A data-driven standardised generalisable methodology to validate a large energy performance Certification dataset: A case of the application in Ireland, Energy and Buildings Volume 323, 2024, 114,774, ISSN 0378–7788, 10.1016/j.enbuild.2024.114774. [4]

## 1 Value of the Data

• EPCs provide valuable big-data on the energy performance and characteristics of buildings. However, the raw EPC database, containing 40 manual entries per dwelling assessment, contains erroneous and outlier data that can skew analysis. This filtered EPC dataset removes erroneous entries, and outliers, improving the quality and robustness of this freely available data source.
• Processing raw EPC data, with over a million data points in 2023 is a laborious task required before using it as input for building stock models. Provision of a cleaned, filtered EPC dataset relieves stakeholders interacting with the dataset allows stakeholders to turn data more quickly into quality information while standardisation by expert users improves data quality.
• The dataset was processed using the rigorous, reproducible, adaptable and evolving methodology outlined in Raushan et al. (2024) [4], that importantly evolves as the database evolves. This is achieved through automated analysis of data features before selection of an appropriate data method. As the characteristic of the data evolves, so does the outlier method.
• This standardised, data-driven filtering approach is generalisable to EPC databases from other countries/regions to improve data quality.
• The standardised, automated, data-driven, adaptive methodology developed in this research will serve as a robust tool for data validation, facilitating the swift transformation of data into actionable information leading to contemporaneous climate policy. The resulting dataset, readily available, filtered using this methodology can benefit energy analysts, financial institutions, policymakers, and the broader building stock research community by providing a higher quality, pre-processed foundation for analysis and modelling.

## 2 Background

The motivation for compiling this dataset stems from the need to improve the quality and reliability of Energy Performance Certificate (EPC) data for building stock analysis in Ireland. Raw EPC datasets often contain inconsistencies, errors, and outliers. This data-driven, standardised methodology was designed to clean and filter the EPC data, ensuring its reliability for further research and policy development for all users. It provides a standard starting place for all stakeholders and democratises access to robust housing stock EPC data. This dataset supports the associated research article by providing a robust, filtered EPC database and complements the original research by offering detailed insights into the dataset creation process*.*

## 3 Data Description

**Fig. 1:**
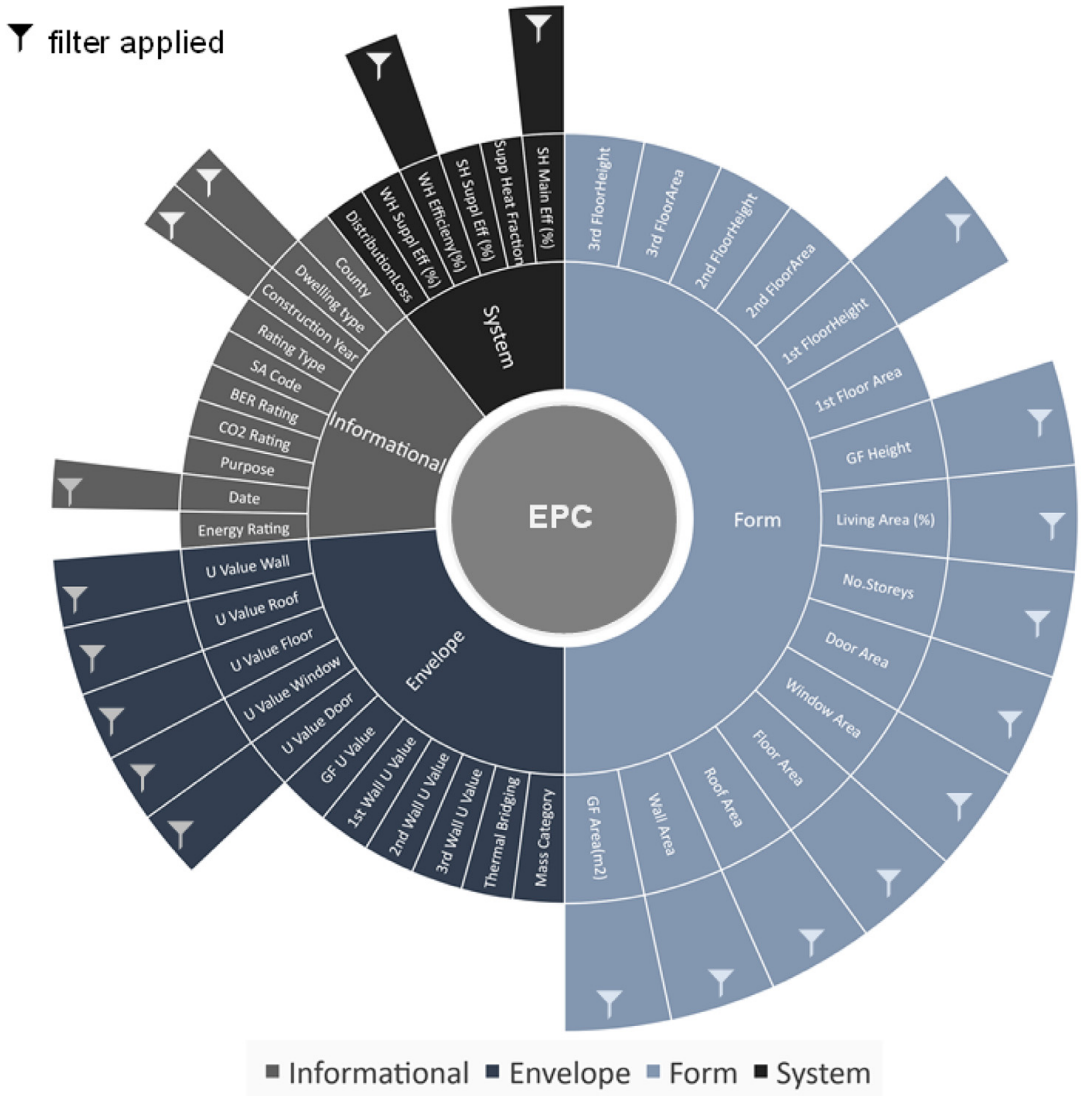
Energy Performance Certificate variables, categorised by properties.

The unfiltered EPC database includes 211 variables describing 1006,000 buildings in Ireland in 2023. This represents approximately ∼50 % of the Irish housing stock. Fig. 1 presents 45 of these variables, categorised as (1) Informational, (2) form, (3) envelope and (4) system. Fig. 1 illustrates the manually inputted variables which were filtered. Table 1 provides further detail on any additional data segmentation applied within individual variables, and the results of upper and lower bounds for each segmented variable filter applied to the BER database.

**Table 1:** Data segmentation and variable filter results.

Filter ID		*Filter Name*	Segmentation Type	Fences	
				Lower	Upper
**1**		***TypeofRating***	No	Provisional removed	
**2**		***UValueWall***	Age		
	i	*Pre-thermal building regulation (≤ 1977)*		0.20	2.40
	ii	*Post-thermal building regulation (≥ 1978)*		0.14	1.10
**3**		***UValueRoof***			
	i	*Pre-thermal building regulation (≤ 1977)*		0.13	2.30
	ii	*Post-thermal building regulation (≥ 1978)*		0.11	0.68
**4**		***UValueFloor***			
	i	*Pre-thermal building regulation (≤ 1977)*		0.16	1.14
	ii	*Post-thermal building regulation (≥ 1978)*		0.11	1.14
**5**		***UValueWindow***			
	i	*Pre-thermal building regulation (≤ 1977)*		1.18	5.70
	ii	*Post-thermal building regulation (≥ 1978)*		0.77	4.80
**6**		***UValueDoor***			
	i	*Pre-thermal building regulation (≤ 1977)*		1.10	3.90
	ii	*Post-thermal building regulation (≥ 1978)*		0.83	3.54
**7**		***WallArea***	Dwelling Type		
	i	*Semi-detached house*		53.19	147.96
	ii	*End of terrace house*		56.62	155.61
	iii	*Detached house*		31.68	336.19
	iv	*Top-floor apartment*		4.63	139.35
	v	*Mid-terrace house*		29.24	163.64
	vi	*Maisonette*		20.88	255.43
	vii	*House*		57.96	392.64
	viii	*Apartment*		0.03	135.15
	ix	*Ground-floor apartment*		5.79	113.86
	x	*Mid-floor apartment*		0.75	104.89
	xi	*Basement Dwelling*		0.94	148.01
**8**		***RoofArea***			
	i	*Semi-detached house*		32.34	127.39
	ii	*End of terrace house*		29.99	108.82
	iii	*Detached house*		30.08	290.85
	iv	*Top-floor apartment*		12.16	126.58
	v	*Mid-terrace house*		32.54	110.68
	vi	*Maisonette*		1.43	84.78
	vii	*House*		0.00	284.29
	viii	*Apartment*		0.00	725.23
	ix	*Ground-floor apartment*		0.19	214.01
	x	*Mid-floor apartment*		0.00	0.00
	xi	*Basement Dwelling*		0.21	238.13
**9**		***FloorArea***			
	i	*Semi-detached house*		31.13	119.09
	ii	*End of terrace house*		29.97	104.74
	iii	*Detached house*		28.87	255.60
	iv	*Top-floor apartment*		0.00	0.00
	v	*Mid-terrace house*		31.65	101.19
	vi	*Maisonette*		3.85	841.00
	vii	*House*		8.56	248.01
	viii	*Apartment*		1.41	1596.88
	ix	*Ground-floor apartment*		2.23	107.71
	x	*Mid-floor apartment*		0.00	0.00
	xi	*Basement Dwelling*		0.00	141.22
**10**		***WindowArea***			
	i	*Semi-detached house*		7.09	41.99
	ii	*End of terrace house*		5.66	34.32
	iii	*Detached house*		5.61	83.48
	iv	*Top-floor apartment*		2.02	38.71
	v	*Mid-terrace house*		4.29	29.02
	vi	*Maisonette*		1.83	35.75
	vii	*House*		5.44	78.07
	viii	*Apartment*		1.72	47.62
	ix	*Ground-floor apartment*		2.96	32.76
	x	*Mid-floor apartment*		2.77	41.77
	xi	*Basement Dwelling*		0.00	32.29
**11**		***Door + Window Area***	No	NA	NA
**12**		***Ground Floor Area***	Dwelling Type		
	i	*Semi-detached house*		48.75	186.83
	ii	*End of terrace house*		58.68	190.49
	iii	*Detached house*		52.24	422.20
	iv	*Top-floor apartment*		18.29	153.06
	v	*Mid-terrace house*		61.13	189.89
	vi	*Maisonette*		12.82	182.30
	vii	*House*		53.63	453.75
	viii	*Apartment*		14.73	143.84
	ix	*Ground-floor apartment*		14.74	125.67
	x	*Mid-floor apartment*		8.61	114.22
	xi	*Basement Dwelling*		2.43	189.37
**13**		***DoorArea***			
	i	*Semi-detached house*		0.00	5.86
	ii	*End of terrace house*		1.54	17.97
	iii	*Detached house*		0.00	6.28
	iv	*Top-floor apartment*		0.00	1.91
	v	*Mid-terrace house*		1.61	21.04
	vi	*Maisonette*		1.83	6.21
	vii	*House*		0.00	4.73
	viii	*Apartment*		1.78	2.02
	ix	*Ground-floor apartment*		1.45	2.40
	x	*Mid-floor apartment*		0.00	1.91
	xi	*Basement Dwelling*		0.29	2.23
**14**		***LivingAreaPercent***			
	i	*Semi-detached house*		11.28	35.17
	ii	*End of terrace house*		12.97	59.49
	iii	*Detached house*		7.55	45.89
	iv	*Top-floor apartment*		0.24	62.29
	v	*Mid-terrace house*		13.03	72.13
	vi	*Maisonette*		6.40	78.29
	vii	*House*		6.62	39.73
	viii	*Apartment*		4.39	57.59
	ix	*Ground-floor apartment*		3.73	64.30
	x	*Mid-floor apartment*		21.59	65.04
	xi	*Basement Dwelling*		4.70	81.83
**15**		***GroundFloorHeight***	No	2.40	3.37
**16**		***HSMainSystemEfficiency***	System type		
	i	*Conventional Heating system*		23.07	94.61
	ii	*Heat pumps*		174.93	635.34
**17**		***WHMainSystemEff***	System type		
	i	*Conventional Heating system*		24.33	94.74
	ii	*Heat pumps*		127.26	350.74
**18**		***CountyName***	No	Non-existent locations removed	

## 4 Experimental Design, Materials and Methods

**Fig. 2:**
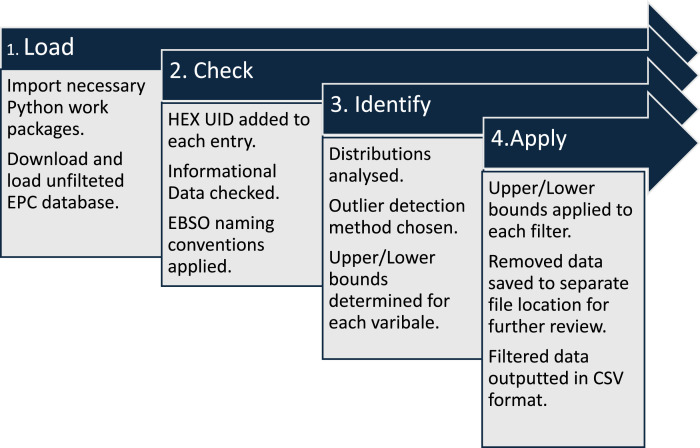
EPC filtering, methodological design, and process.

This section outlines the datasets and scripts included with this article. It also provides a detailed description of the steps required download, process, and generate a filtered EPC dataset. The unfiltered EPC dataset included 1006,000 records in 2023, providing data on dwellings which we have divided into four separate categories: (1) Informational, (2) Form, (3) Envelope, and (4) System. Different methods were required to process each of these four categories and data types. A set of scripts were used, utilising Python, to analyse and process the unfiltered EPC dataset. The scripts (included) can be accessed using different Integrated Development Environments (IDEs), Visual Studio Code was used in this analysis to execute the scripts. The filtered dataset is also included with this article. Fig. 2 provides a high-level description of the methodological design while steps 1 to 4 provide a detailed description of each section of the script required to replicate this methodology. Specific lines of code are referenced using the “Execution order” used in the associated Python_Processing_Script.ipynb script [5].

1. **Load:** This step imports the required Python work packages and loads the unfiltered EPC dataset into the script dataframe. The Sustainable Energy Authority of Ireland (SEAI) host the national (unfiltered) EPC dataset. This dataset is freely available and accessible online.
  1.1. Download the unfiltered EPC dataset in tab delimited (.txt) format from SEAI using the link provided (https://ndber.seai.ie/BERResearchTool/ber/search.aspx).
  1.2. Note the folder location of where the unfiltered file is saved.
  1.3. Using Visual Studio (VS), or alternative IDE of choice, update the folder location in execution order 2 in Python_Processing_Script.ipynb with this saved location from step 1.2. In this study, VS is used to execute a Jupyter Notebook (ipynb) using a python kernel.
  1.4. Execute Python_Processing_Script.ipynb using the “Run All” command in Visual Studio.
  1.5. Necessary python libraries are loaded automatically, including “pandas (Version: 1.3.4)”, “numpy (Version: 1.21.2)”, “pyplot (Version: 3.4.3, module of Matplotlib package)”, “statistics (Version: 1.0.3.5)”, “sklearn (Version 1.4.2, module of scikit-learn package)”, “plotly (Version: 5.13.0)”, and “mpl_toolkits (Version: 3.4.3, module of Matplotlib package)”. Tested versions of each package/module included in parentheses.
2. **Check:** This section of the Python_Processing_Script.ipynb script adds an essential HEX UID column to the dataset and processes the informational data to check for consistency errors. This step does not remove any erroneous data but instead creates a copy of the column and updates the entries to be consistent with the remainder of the dataset e.g., the “County Name” column is duplicated and then updated to provide a consistent naming format across all counties.
  2.1. Unique HEX ID (HEX UID) is added to each entry in the dataset.
  2.2. Python_Processing_Script.ipynb script checks informational data relating to dwelling location (ensuring locations exist), date of assessment (which are not in the future, or which were conducted before the establishment of EPC programme), year of construction (which are not in the future), energy rating, and CO2 rating (negative CO2 ratings identified).
  2.3. EPC Variable names are updated to correspond with the European Building Stock Observatory standard naming convention.
3. **Identify:**
  3.1. Using the statistical python packages identified in section 1.5, the distribution of each selected variable shown in Table 1 is identified. The best-suited method to analyse each distribution type is then used, based on the selection process shown in Table 2.

**Table 2:** Distribution characteristics and filter rule applied to identify outliers.

Distribution type	Characteristics of distribution	Rule applied
Continuous	normal or quasi normal distribution	boxplot method
	non-normal distribution and extreme values on both end	
	Skewed distribution	adjusted boxplot
Multi-modal	multi-modal distribution	segmentation
	segmented distributions are normal or quasi normal	boxplot method
	segmented distributions are non-normal with extreme values on both end	Data trimming
	segmented distributions are skewed	adjusted boxplot
Categorical	categorical and entries are not machine readable	standardise input values using validated dictionaries
  3.2. Outliers are detected based on distributional characteristics and analysed using statistical techniques suited to the nature of each variable. For example, the dataset is segmented by features like building type and construction period (pre- and post-thermal building regulation), with tailored bounds set according to normal, skewed, or multimodal distributions as relevant [4]. This process identifies data points that deviate significantly from typical values within each category, reducing the likelihood of misclassifying legitimate data as outliers.
4. **Apply:**
  4.1. Outlier limits are applied to all relevant variables, see Table 1.
  4.2. All data which is identified as an outlier or erroneous is saved to a separate file (outlier_erroneous_data.csv) for user review. This file is saved to the original save location identified in step 1.2.
  4.3. All filtered data is outputted in csv format to the original save location identified in step 1.2 and titled “filtered.csv”. The filtered dataset serves as a representative foundation for modelling a building stock, improving data reliability and applicability.

## Limitations

One limitation of the output data arises from the segmentation based on feature distributions, which can reduce the number of available data points in certain categories. For instance, the “Ground Floor Area” and “Roof Area” produced inconsistent results for segments like “basement dwellings” and “houses” due to limited data points. Additionally, discrepancies in U-values for roof and floor elements could not always be definitively classified as errors, reflecting gaps in data precision. However, as the dataset expands, these limitations are expected to decrease, improving the reliability of future analyses.

## Ethics Statement

The authors have read and follow the ethical requirements for publication in Data in Brief and confirm that the current work does not involve human subjects, animal experiments, or any data collected from social media platforms.

## CRediT Author Statement

**Kumar Raushan**: Conceptualization, Methodology/Study design, Software, Validation, Formal analysis, Investigation, Data curation, Writing – original draft, Writing – review & editing, Visualization, Project administration. **Tomás Mac Uidhir**: Conceptualization, Methodology/Study design, Software, Validation, Formal analysis, Investigation, Data curation, Writing – original draft, Writing – review & editing, Visualization, Project administration. **Marisa Llorens**: Conceptualization, Methodology/Study design, Software, Validation, Formal analysis, Investigation, Data curation, Writing – original draft, Writing – review & editing, Visualization, Project administration. **Brian Norton:** Conceptualization, Validation, Writing – original draft, Writing – review & editing, **Ciara Ahern:** Conceptualization, Methodology/Study design, Software, Validation, Formal analysis, Investigation, Data curation, Writing – original draft, Writing – review & editing, Visualization, Project administration

## Data Availability

Mendeley DataDataset: A generalisable data-driven filtering methodology for Energy Performance Certification databases (Original data). GithubPython_Processing_Script (Original data). SEAI NBERBER Public Search (Reference data).
